# Rat Olfactory Mucosa Mesenchymal Stem/Stromal Cells (OM-MSCs): A Characterization Study

**DOI:** 10.1155/2020/2938258

**Published:** 2020-01-29

**Authors:** Rui D. Alvites, Mariana V. Branquinho, Ana R. Caseiro, Irina Amorim, Sílvia Santos Pedrosa, Alexandra Rêma, Fátima Faria, Beatriz Porto, Cláudia Oliveira, Paula Teixeira, Rui Magalhães, Stefano Geuna, Artur S. P. Varejão, Ana C. Maurício

**Affiliations:** ^1^Departamento de Clínicas Veterinárias, Instituto de Ciências Biomédicas de Abel Salazar (ICBAS), Universidade do Porto (UP), Rua de Jorge Viterbo Ferreira, nº 228, 4050-313 Porto, Portugal; ^2^Centro de Estudos de Ciência Animal (CECA), Instituto de Ciências, Tecnologias e Agroambiente da Universidade do Porto (ICETA), Rua D. Manuel II, Apartado 55142, 4051-401 Porto, Portugal; ^3^REQUIMTE/LAQV – U. Porto – Porto/Portugal, Departamento de Engenharia Metalúrgica e Materiais, Faculdade de Engenharia, Universidade do Porto, Rua Dr. Roberto Frias, s/n, 4200-465 Porto, Portugal; ^4^Escola Universitária Vasco da Gama (EUVG), Avenida José R. Sousa Fernandes, nº 197 Lordemão, 3020-210 Coimbra, Portugal; ^5^Departamento de Patologia e Imunologia Molecular, Instituto de Ciências Biomédicas de Abel Salazar (ICBAS), Universidade do Porto (UP), Rua de Jorge Viterbo Ferreira, nº 228, 4050-313 Porto, Portugal; ^6^i3S – Instituto de Investigação e Inovação em Saúde, Universidade do Porto, R. Alfredo Allen, 4200-135 Porto, Portugal; ^7^Institute of Molecular Pathology and Immunology of the University of Porto (IPATIMUP), 4200-465 Porto, Portugal; ^8^Laboratório de Citogenética, Instituto de Ciências Biomédicas de Abel Salazar (ICBAS), Universidade do Porto (UP), Rua de Jorge Viterbo Ferreira, nº 228, 4050-313 Porto, Portugal; ^9^Universidade Católica Portuguesa, CBQF – Centro de Biotecnologia e Química Fina – Laboratório Associado, Escola Superior de Biotecnologia, Rua Arquiteto Lobão Vital 172, 4200-374 Porto, Portugal; ^10^Department of Clinical and Biological Sciences, and Cavalieri Ottolenghi Neuroscience Institute, University of Turin, Ospedale San Luigi, 10043 Orbassano, Turin, Italy; ^11^Departamento de Ciências Veterinárias, Universidade de Trás-os-Montes e Alto Douro (UTAD), Quinta de Prados, 5001-801 Vila Real, Portugal; ^12^CECAV, Centro de Ciência Animal e Veterinária, Universidade de Trás-os-Montes e Alto Douro (UTAD), Quinta de Prados, 5001-801 Vila Real, Portugal

## Abstract

Stem/stromal cell-based therapies are a branch of regenerative medicine and stand as an attractive option to promote the repair of damaged or dysfunctional tissues and organs. Olfactory mucosa mesenchymal stem/stromal cells have been regarded as a promising tool in regenerative therapies because of their several favorable properties such as multipotency, high proliferation rate, helpful location, and few associated ethical issues. These cells are easily accessible in the nasal cavity of most mammals, including the rat, can be easily applied in autologous treatments, and do not cope with most of the obstacles associated with the use of other stem cells. Despite this, its application in preclinical trials and in both human and animal patients is still limited because of the small number of studies performed so far and to the nonexistence of a standard and unambiguous protocol for collection, isolation, and therapeutic application. In the present work a validation of a protocol for isolation, culture, expansion, freezing, and thawing of olfactory mucosa mesenchymal stem/stromal cells was performed, applied to the rat model, as well as a biological characterization of these cells. To investigate the therapeutic potential of OM-MSCs and their eventual safe application in preclinical trials, the main characteristics of OMSC stemness were addressed.

## 1. Introduction

In the last decades, cell-based therapies have stood out in the medical and research fields, appearing as an alternative to the treatment of several diseases and pathologies previously difficult to approach [1]. The application of these therapies is based on the repair of the mechanisms associated with the beginning, establishment, or progression of the disease. Through trophic effects or native cell replacement [2], cell therapies use stem/stromal cells to promote their differentiation in specific locations and under intended pathological conditions [3]. Stem/stromal cells are classified as undifferentiated, capable of proliferating indefinitely under proper conditions and able to differentiate into cell types and tissues depending on the stimulus received. Over the years, the search for readily available, safe, stable, and potentially effective stem/stromal cells for regular use in regenerative medicine has been intense [4]. These characteristics were initially identified in cells isolated from the mouse bone marrow, which exhibited desirable characteristics such as plastic adhesion and modification into fibroblastic colony units under culture [5]. Developing from the mesoderm and with ability to differentiate into specialized cells, these cells were later named as mesenchymal stem/stromal cells (MSCs). Also known as multipotent cells, MSCs are heterogenic stromal cells that have already been identified and can be collected in virtually all adult tissues of several species. Able to self-renewing, multipotent, almost always easily accessible, expandable *in vitro *cultures and exceptionally stable from a genetic point of view, they are a fundamental focus of regenerative medicine and tissue repair [6, 7]. The application of MSCs in regenerative medicine deals with special characteristics such as their easy expansion in culture, ability to differentiate into the desired cell types, specific immunological characteristics (immune-privileged and immune-modulation), tropisms for lesion sites, trophic stimulation capacity, and also modulation of tissue functions and inflammation by secreting important bioactive molecules [8]. In contrast to other therapeutic approaches where a complete and unequivocal characterization of the mechanisms of action are stablished before its use, in the case of MSCs, its therapeutic advantages have already been broadly characterized even if there is not yet a complete understanding of its way of action or in vivo function [7].

Since its first description, MSCs have already been identified in several adult tissues, with very few exceptions. Tissues where they have been identified include adipose tissue, amniotic fluid, amniotic membrane, sub-amniotic umbilical cord lining membrane, Wharton's jelly, endometrium, menstrual blood, peripheral blood, placenta and fetal membrane, dental pulp, salivary glands, skin and foreskin, synovial membrane and olfactory mucosa (OM) [4, 9].

Despite its use and regular characterization, there is no universal definition or conclusive assays that allow the unambiguous identification of MSCs in a mixed population of cells [6]. In 2006, the International Society for Cellular Therapy (ISCT) focused on creating a more specific definition of MSCs and establishing minimum criteria for their identification. These cells must: (a) be plastic-adherent under standard culture conditions; (b) present a set of markers (differentiation clusters) including CD73, CD90, CD105 absence of CD14, major histocompatibility complex- (MHC-) II/human leukocyte antigen- (HLA-) DR and markers for hematopoietic lineage such as CD34, CD45; and (c) be able to differentiate *in vitro* in at least three cell lines: adipogenic, chondrogenic, and osteogenic [10]. These characteristics are well-defined for human MSCs, even if slight differences in MSCs isolated from distinct tissues can be identified. Nevertheless, these criteria may not be adequate to characterize MSCs for all species. Commonly used antibodies do not recognize the analogous surface antigens of animal cells with the same affinity, and variations in expression levels thereof may occur as compared to the manifestations in human cells [11]. However, the criteria defined for humans are still those used for the characterization of animal cells and should be used in an adapted and weighted manner. Concerning the capacity for differentiation, multiple studies carried out have made it possible to perceive that MSCs are capable not only of traditional tridifferentiation but also of originating other cells and tissues with mesodermal (ligaments, tendons, cardiomyocytes, muscles), ectodermal and endodermal origin (skin, retina, lungs, hepatocytes, renal tubes, pancreatic islets, sebaceous glands and ducts and neural cells) [12]. Also, recently new markers have been explored to identify those that can be considered stemness-associated MSC stromal cell markers, in opposition to the traditional MSC markers that some authors question and indicate as more appropriate to be considered stromal cell markers [13]. In this group, CD271 is indicated as a potential precursor for homogeneous subpopulations of MSCs and described as a way to improve culture homogeneity. Even so, some studies show that even CD271-MSCs are heterogeneous in their proliferative, differentiation and immunomodulatory potential, contributing to the heterogeneous adult MSC properties [14]. Thus, the identification of new functionally relevant surface markers is important to ensure the creation of robust quality criteria that will allow better control in the use of MSCs.

MSCs collected at the *lamina propria* of OM are named olfactory mucosa mesenchymal stem/stromal cells (OM-MSCs) and although the elements of the olfactory system originate from an interaction between ectoderm-derived placodes and migrating cranial neural crest cells [15] *lamina propria* components and therefore OM-MSCs are thought to be largely derived from the neural crest [16]. OM-MSCs were initially identified in the OM of an embryonic rat [17]. The different studies already carried out for its characterization allowed to identify its capacity to form fibroblastic-like low density colonies and the expression of classic markers and those related to differentiation [15]. Although able to tridifferentiate, OM-MSCs seem to differentiate more easily into osteoblasts than into chondroblasts and adipocytes [15]. In addition, they can still follow a myogenic and neurogenic differentiation [18] and its conditioned medium promotes the proliferation of ensheathing cells, oligodendrocyte precursor cells and myelination *in vitro* [18, 19]. The most important characteristics that make these cells optimal candidates for regenerative medicine are its high versatility, wide distribution in nasal cavity [20], easy access with few associated ethical issues and little susceptibility to the development of chromosomal or tumorigenic alterations [21, 22]. Even with high mitotic activity, these cells can maintain self-renewal ability in culture for long periods of time by conserving telomeric activity and inhibiting apoptotic activity, characteristics that are not affected by the donor's age [23].

The anatomical location of the olfactory mucosa is excellent for both *antemortem* and *postmortem* collection in most species of clinical interest. OM harvesting in small animal models, such as rat, mouse, or rabbit, requires euthanasia, but the peripheral location of the tissue and use of appropriate aseptic techniques during collection allow to reuse animals from other studies, once there is a guarantee that no contamination of the olfactory region occurred [17, 24–26]. An *antemortem* collection method for rats have also been described, but its complexity makes it harder to be applied regularly to collect OM and isolate OM-MSCs [24, 27] In humans and larger species, OM can be easily collected *in vivo* using rhinoscopy techniques and nasal forceps [15, 28–30], then making OM-MSCs great candidates for autologous transplantation in domestic animals and humans to treat different types of lesions, specifically nerve damages.

Previous studies on OM-MSCs' secretome allowed to identify the production and secretion of bioactive molecules with a direct influence on neural differentiation, namely in the production and maturation of glial cells [31]. In addition, its clinical potential has already been tested in the field of experimental neurology, as in the treatment of neurodegenerative diseases of the central nervous system [32], hippocampal lesions [33, 34], in the regeneration of the peripheral nerves and cranial nerves [35–38] and in cases of spinal cord trauma [39, 40]. Its immune-suppressive effect over autoimmune diseases [41, 42] and regenerative promotion in myocardial tissue after infarct [43] and ischemic tissues [44] was also evaluated.

So far, OM-MSCs have been studied and characterized in different extents in species such as humans [15], mouse [45], rabbit [26], dog [28], sheep, horse, macaque, and lemur [29]. Rat's OM-MSCs, an animal model with a particular interest in regenerative medicine of the nervous system and peripheral nerve, have already been explored in some studies [17, 27, 29], although there is still much to discover and study about OM-MSCs in this species.

The aim of this work was to perform a complete characterization of rat's OM-MSCs, using a complete, longitudinal, and sequential approach not observed in other studies. Special emphasis was given to the methods of cell isolation, culture and expansion, freezing and thawing protocols. Moreover, its biological characterization was carried out in relation to cell behavior in culture, determination of cellular karyotype, determination of cell genetic expression, identification of specific proteins from the cell surface, testing of multilineage differentiation capacity and characterization of the secretome profile during expansion, regarding immune-modulatory capacity. Envisioning their subsequent use in regenerative therapies, the OM-MSC stemness characteristics were also evaluated.

## 2. Materials and Methods

All procedures performed on animals were approved by the Organism Responsible for Animal Welfare (ORBEA) of the Abel Salazar Institute for Biomedical Sciences (ICBAS) from the University of Porto (UP) (project 209/2017) and by the Veterinary Authorities of Portugal (DGAV) (project DGAV: 2018-07-11 014510). All animal testing procedures were in conformity with the Directive 2010/63/EU of the European Parliament and the Portuguese DL 113/2013, and followed in accordance to the OECD Guidance Document on the Recognition, Assessment and Use of Clinical Signs as Humane Endpoints for Experimental Animals Used in Safety Evaluation (2000). Adequate measures were taken to minimize pain and discomfort considering humane endpoints for animal suffering and distress.

Ten Rats (*Rattus norvegicus*), Sprague Dawley, male gender, with 8-9 weeks of age and 200–300 g BW were used for the collection of the OM tissue. In a 3 R's perspective, and in collaboration with other works within the research group, animals considered for tissue collection were reused, as they were sacrificed for other research purposes. Animals were preanesthetized with xylazine (Rompun®, 1.25 mg/g) and Ketamine (Imalgene 1000®, 9 mg/100 g) intraperitoneally in a single administration. Euthanasia was performed using a chemical method of anesthetic overdosing with pentobarbital sodium (Eutasil® 200 mg/ml injectable solution, Ceva Santé Animale, 200 mg/kg) administered intraperitoneally.

### 2.1. Collection of Olfactory Mucosa

OM was collected as previously described [24]. The collection itself was made using a tweezer, and atraumatically, the OM lining the olfactory recess, regions of the nasal septum and remnant ethmoturbinates was detached and immediately immersed in transport medium consisting on phosphate-buffered saline solution (PBS) (DPBS, Sigma-Aldrich®). A needle was used to gently peel the mucosa from the tip of the tweezers for deposition into the solution. OM pooled samples from different animals was collected and conditioned at 4–6°C.

### 2.2. Sample Transport and Storage

Once the collection of OM was completed, the fragments were transported in the transport medium to the laboratory facilities as soon as possible under refrigerated temperatures of 4°C–6°C. Here, whenever immediate processing was not possible, fragments of OM were centrifuged within the transport medium, with subsequent removal of the supernatant. The fragments were then resuspended in a storage solution comprising 3% antibiotics [penicillin 100 U/mL/streptomycin 100 *μ*g/mL) (Sigma-Aldrich®)], 3% antimycotic [amphotericin B (2.5 *μ*g/mL) (Sigma-Aldrich®)] and PBS. After this procedure, the fragments were stored overnight with the storage solution at a refrigeration temperature of 4°C–6°C or cryopreserved for later use. To ensure integrity, the time between this storage and the processing of the samples never exceeded 24–48 h after euthanasia and OM collection, excluding when the tissue was cryopreserved. All cells used and considered in the different assays of this work originated from the pool obtained from the 10 rats from which OM was collected.

### 2.3. Isolation and *In Vitro* Expansion of OM-MSCs

All procedures related to cell isolation and expansion were performed under strict aseptic conditions, within the laminar flow hood. OM fragments were centrifuged and supernatant eliminated. OM fragments were carefully transferred to a sterile Petri dish and fragments immersed in PBS to avoid dehydration. OM fragments were dissected into smaller pieces, reducing them to portions no larger than 1-2 mm^2^. Two phases were considered during the isolation method. In the first phase, an enzymatic digestion was performed to release OM-MSCs from the extracellular matrix. The reduced fragments were transferred to a tube, and a solution of 0.25% Trypsin-EDTA (Sigma-Aldrich®) for enzymatic digestion was added. Enzymatic digestion was performed in a water bath (37°C) for approximately 15 min, in constant agitation. After enzymatic digestion, the fragments were centrifuged and supernatant eliminated. Secondly, the fragments were transferred to 6-well tissue culture treated plates (Multiwell Cell Culture Plates 6 Wells, VWR®) at a density of 1–3 fragments per well, letting them stand for 5–10 minutes to allow adherence to the plastic surface [25]. Once adherence was confirmed, preformulated culture medium consisting of Basal Medium (DMEM/F12 + GlutaMAX™ supplement, Gibco®) supplemented with 10% certified Fetal Bovine Serum (FBS) (One Shot Fetal Bovine Serum, Gibco®), 1.5% Penicillin-Streptomycin and 1.5% Amphotericin B [26], in sufficient volume to cover the explants, was added to each well, carefully, so as not to disturb explants adhered to the plastic surface. Where floating fragments were identified (not adhered), the medium was removed and the process repeated minutes later. The plates were then incubated in standard conditions (37°C, 5% CO_2_ humidified atmosphere). Wells were observed daily with an inverted phase contrast microscope (Axiovert 40 CFL, Zeiss®) in search for OM-MSCs radiating from the explants. Media were refreshed every 2-3 days.

After one week, cells were observed radiating from the explants and invading the wells of the plate. About 4 days later, the cells reached an intended confluence of 70–80%. At this stage, the medium and the explants were carefully removed. Each well was washed abundantly with PBS, and cells were detached using 0.25% Trypsin-EDTA solution and an incubation of 3–5 min under standard conditions. The contents of all wells were then collected and centrifuged (1600 rpm, 10 min), the supernatant eliminated and cells reseeded in a T75 culture flask (Nunc™ EasyFlask™ 75 cm^2^, ThermoScientific®) at a density of 2.1 × 10^6^ cells/cm^2^.

### 2.4. Cell Behavior in Culture

#### 2.4.1. Growth Curve and Cell Viability

Cells were maintained in culture over 35 days to determine their growth curve. Whenever a confluence of 70–80% was reached, a new cell passage was made, resulting in a total number of 15 passages. Each passage was performed by elimination of culture medium, washing with PBS, cell detachment with 0.25% Trypsin-EDTA solution and incubation during 3–5 min under standard conditions, centrifugation (1600 rpm, 10 min) and elimination of supernatant. To determine the number of cells and their viability at each passage, the Trypan blue exclusion cell assay [46], with counting of cells with an automatic counter (Countess II FL Automated Cell Counter, Thermo Fisher Scientific®), was performed.

#### 2.4.2. Population Doubling Time (PDT)

OM-MSCs used to calculate PDT were thawed, maintained in culture, and harvested at 70–80% confluence. Then, they were plated with culture medium in 10 wells of a 12-well plate (12 Flat Test plate, Orange Scientific®) at a density of 0.05 × 10^6^ cells/cm^2^. For 10 days, the number of cells from one well per day was determined. At the end of the 10 days, the PDT was defined through the method proposed by Lotfy et al. [47]. The formula used was PDT = CT/PDN, being CT the culture time (in this case, 10 days) and PDN the population doubling number. The PDN was calculated using the formula PDN = 3.32(log Xf − log Xi) + *S*, being Xf the cell harvested at the end of the subculture, Xi the cell number used as inoculum and *S* the doubling level of the inoculum used to start the subculture being quantified. Here, as the value of *S* is unknown, it was considered as 0. PDT was determined for 3 different moments, namely cells in P2-P3, P6-P7, and P13-P14. For each passage, triplicates were considered.

#### 2.4.3. Colony Forming Unit Assays

Colony Forming Units (CFUs) assay was performed according to the method indicated by Penfornis et al. [48]. Briefly, OM-MSCs at P3 were maintained in culture until a confluence of 70–80%, followed by enzymatic detachment with 0.25% Trypsin-EDTA solution. Collected cells were diluted using a 1 : 2 serial dilution in growth medium to allow the plating of a density ranging from 10 to 80 cells in each well of a six-well plate. Cells were incubated for 14 days under standard conditions with daily monotorization to confirm the development of colonies. After this period, culture medium was removed and wells were stained with 0.5% (v/v) of Crystal Violet solution for 5–10 min at room temperature. The number of colonies in each well was quantified using a magnifying glass (Leica Zoom 2000, Meyer instruments®). Only visible colonies with more than 1 mm of diameter or with over 50 cells were considered. Overlapping colonies were not considered. The clonal efficiency (% of clonogenicity) was calculated using the formula (mean number of colonies counted/total number of seeded cells) × 100. To establish the CFU value, six wells (*N* = 6) from a six-well plate were counted.

#### 2.4.4. Cryopreservation and Thawing

OM tissue fragments, as well as OM-MSCs isolated and at different passages were subjected to cryopreservation and thaw cycles to determine the viability, subsequent cell performance and the efficacy of this method for cell and tissue preservation. Cells in culture were subjected to enzymatic detachment with 0.25% trypsin-EDTA solution, collection and automatic counting. OM-MSCs were cryopreserved with culture medium and 10% Dimethyl sulfoxide (DMSO) (SigmaAldrich®) [49] in cryovials with at least 1 × 10^6^ cells. The cryovials were then transferred to a freezing container with Isopropyl alcohol (Nalgene® Mr. Frosty®, ThermoScientific®) for slow freezing (−1°C/minute) at −80°C. After a maximum period of 3 days, the cryovials were transferred to canisters inside a liquid nitrogen container (−196°C) for long-term cryopreservation (LS750 Cryogenic Sample Storage, Taylor Wharton®). The process is identical for cryopreservation of OM fragments, with the difference that in this case the cryovial are placed about 15–20 minutes at 4° to allow penetration of DMSO into the tissue before its transference to −80°C.

The cryovial content was thawed using a water bath (37°C) for fast thawing. Inside the laminar flow hood, the cryovial contents were quickly collected, centrifuged, and the supernatant discarded. OM-MSCs were resuspended in culture medium, counted, cultured, and maintained under standard conditions. The procedure for the OM fragments is identical, the explant plating after enzymatic treatment being performed after thawing.

### 2.5. Differentiation Protocols

For all differentiation protocols, cells in P4 after thawing were used.

#### 2.5.1. Adipogenic Differentiation and Oil Red O Staining

For the adipogenic differentiation protocol, 1 × 10^4^ cells/cm^2^ were seeded in the wells of a 12-well plate, with addition of culture medium. The plate was incubated under standard conditions for 4 days. After this period, the culture medium of 10 wells was replaced by complete adipogenesis differentiation medium (StemPro® Adipogenesis Differentiation Kit, Gibco®) 2 wells were used as controls and maintained with the usual culture medium. Following the manufacturer's instructions, the media were replaced every 3-4 days and the cells maintained in differentiation for 14 days. At the end of this period, Oil Red O staining protocol was performed using a handmade solution. The culture and differentiation media were removed, and the wells were gently washed with PBS. Cells were fixed with 4% formaldehyde (3.7–4% buffered to pH7, reference# 252931.1315, Panreac AppliChem®) for 10 minutes at room temperature, and the wells washed 3 additional times with PBS. Oil Red O solution was added to each well and the plate incubated for 10–20 minutes at room temperature. Oil Red O was discarded, and any excess dye removed by several washes with PBS. PBS was added to each well for visualization. The aim of this assay was the identification of intracytoplasmic lipid vacuoles and their red coloration due to the exposure to the Oil Red O solution.

#### 2.5.2. Chondrogenic Differentiation and Alcian Blue Staining

Thawed OM-MSCs were automatically counted and % cell viability determined. The cells were then centrifuged, supernatant removed, and the pellet resuspended in culture medium to generate a cell suspension with 1.6 × 10^7^ viable cells/ml. To generate micro-mass cultures, 5 *μ*l droplets of the cell suspension were placed in the center of 10 wells of a 96-well plate (Cell culture plates, 96-well, VWR®), in order to induce chondrogenic differentiation. The plate was maintained under standard conditions for 2 hours. After this time, chondrogenic differentiation medium (StemPro® Chondrogenesis Differentiation Kit, Gibco®) was added to 8 wells, as 2 wells were considered as control and to these, usual culture medium was added. Following the manufacturer's instructions, media were replaced every 3-4 days and cells maintained in differentiation for 14 days. At the end of this period, the Alcian Blue staining protocol was performed (Alcian Blue 8GX, Sigma-Aldrich®). The culture and differentiation media were removed, and the wells were gently washed with PBS. Cells were fixed with 4% formaldehyde during 20 minutes at room temperature, and the wells washed 3 additional times with PBS. Alcian Blue solution was added to each well and the plate incubated for 30 minutes at room temperature. Alcian Blue was then discarded and wells were rinsed 3 times with acetic acid 3% (v/v). For neutralization of acidity and for visualization by inverted phase contrast microscopy, distilled water was added to all wells. The final aim of this assay was the identification of chondrogenic aggregates and their coloration in blue due to the exposure to Alcian Blue solution.

#### 2.5.3. Osteogenic Differentiation and Alizarin Red S Staining

For osteogenic differentiation, 8 × 10^3^ cells/cm^2^ were seeded into the wells of a 12-well plate. The plate was maintained under standard conditions for 4 days. After this period, the culture medium of 10 wells was replaced by complete osteogenic differentiation medium (StemPro® Osteogenic Differentiation Kit, Gibco®), and 2 wells were used as controls and maintained with the usual culture medium. Following the manufacturer's instructions, media were replaced every 3-4 days and the cells maintained in differentiation for 21 days. At the end of this period, Alizarin Red S Staining protocol was performed using a commercial solution (Alizarin-Red Staining Solution, Milllipore®). The culture and differentiation media were removed, and the wells were gently washed with PBS. Cells were fixed with 4% formaldehyde for 30 minutes at room temperature, and the wells washed twice with distilled water. 40 mM of Alizarin Red solution was added to each well and the plate incubated for 30 minutes. Alizarin Red was then discarded, and wells were rinsed 3 times with distilled water until supernatant became clear. For visualization by inverted phase contrast microscopy, PBS was added to all the wells. The aim of this essay was to identify calcium containing osteocytes stained red after exposure to alizarin Red solution.

#### 2.5.4. Neurogenic Differentiation

For neurogenic differentiation, 4 × 10^3^ cells/cm^2^ were seeded into the wells of a 12-well plate. The plate was maintained under standard conditions, and media replaced every 3-4 days until a confluence of 70–80% was reached. Media were removed from all wells and 8 of them received neurogenic differentiation medium (MSC Differentiation Medium, PromoCell®), and 2 wells were used as controls and maintained with the usual culture medium. The plate was Incubate for 5 days, and media changed every 48 hours.

### 2.6. Reverse Transcriptase Polymerase Chain Reaction (RT-PCR)

RT-PCR targeting specific genes expressed by undifferentiated OM-MSC cells, in passages 4 and 6 was performed. The expression of 19 genes was analyzed to allow the identification of genes associated with markers included in the minimum criteria for the characterization of cells as MSCs according to the ISCT [10] and markers for differentiation. Three genes used as markers for pluripotent stem cells (NANOG, Oct4, Sox2), four genes used as MSC markers (CD105, CD90, CD73, CD44), two genes used as hematopoietic markers (CD45, CD34), and genes used as markers of differentiations: osteogenic (BSP, Runx2), chondrogenic (Coll II, Aggrecan), adipogenic (Ap-2, AdipoQ), neurogenic (NGF and GDNF), tenogenic (Tenomodulin) and musclogenic (Desmin) were analyzed. GAPDH was used as housekeeping gene. For identification of gene expression, a 96-well plate (Prime PCR Custom Plate 96 Well, Bio Rad Laboratories®) was created with 19 predesigned primers for the indicated genes (Supplementary Material – Table S1).

#### 2.6.1. RNA Isolation and cDNA Synthesis

Isolation of RNA was performed with the Aurum™ total RNA Mini Kit (Bio Rad Laboratories®), according to the manufacturer's instructions. Briefly, a 2 × 10^6^ cells pellet was lysed with a lysis solution, DNA removed with a DNAase I enzyme and eluted with 80 *μ*l of an elution solution. RNA was stored at −80°C for later use. Before cDNA synthesis, the amount and purity of the RNA was determined by UV-spectrophotometry by measuring the A_260_/A_280_ (indicator of protein contamination) and A_260_/A_230_ (indicator of polysaccharide, phenol, and/or chaotropic salt contamination) absorbance in a Nanodrop spectrophotometer (Implen, Isaza). Purity values considered acceptable range from 2–2.2 to A_260_/A_280 _and from 1.8–2.2 to A_260_/A_230_ [50].

The first strand cDNAs were synthesized from 3.51 *μ*l of total RNA in 20 *μ*l final volume, using the iScript™ cDNA Synthesis Kit (Bio Rad Laboratories®) according to the manufacturer's instructions. The complete reaction mix was incubated in the thermal cycler (T100™ Thermal Cycler, Bio Rad Laboratories®) using the manufacturer's time and temperature guidelines for the aforementioned kit.

#### 2.6.2. Quantitative RT-PCR Assay

RT-PCR assay was performed using the CFX96 Touch™ Real-Time PCR Detection System (Bio Rad Laboratories®) under standard PCR conditions and using itaq™ Universal SYBR Green Supermix (Bio Rad Laboratories®) according to the manufacturer's guidelines. Plates were read in the Real-Time PCR Detection System. Each pair of primers targeting the genes was used to analyze their expression in OM-MSCs. Plates containing the mix targeting the 19 genes were subjected to the temperature cycles indicated by the manufacturers. Once the RT-PCR was finished, the gene expression was analyzed. To confirm the specificity of the product, an analysis of the melting curve was also performed.

The values of threshold cycle (Ct) <29 are considered strong positive reactions indicative of abundant target nucleic acid in the sample; Ct between 30 and 39 are considered positive reactions indicative of moderate amounts of target nucleic acid; Ct >39 correspond to weak reactions and indicate minimum target nucleic acid values or environmental contamination. For each passage, the ΔCt value was calculated using the formula ΔCt = Ct_target gene_ − Ct_housekeeping gene_. Fold differences between the two passages were calculated using the standard ΔΔCt with the formula ΔΔCt = ΔΔCt_P6_ − ΔΔCt_P4_. The relative quantification (RQ) was calculated by the formula: RQ = 2^−(ΔΔCt)^ Genes with RQ values <0.5 are down-regulated and with RQ values >2 are up-regulated.

### 2.7. Cytogenetic Analysis

For evaluation of the karyotype, OM-MSCs in three different passages (P5, P8 and P11) were submitted to cytogenetic analysis to determine chromosomal stability in terms of chromosome number and occurrence of neoplastic changes. For all passages, 70–80% confluence was reached, and culture medium was changed and supplemented with 10 *μ*g/ml colcemid solution (KaryoMAX® Colcemid™ Solution, Gibco®). After 4 h, the OM-MSCs were collected and resuspended in 8 mL of 0.075 M KCl solution, followed by incubation under standard conditions for 15 minutes. After a centrifugation (1700 rpm), 8 ml of ice-cold fixative comprising methanol and glacial acetic at a proportion of 3 : 1, was added and mixed together and the cells were centrifuged again. Three fixation rounds were carried out. After the last centrifugation, the suspension of OM-MSCs was spread over glass slides. Karyotype analysis was performed by one scorer on Giemsa-stained cells. For the different passages, a specific number of cells in metaphase were evaluated dependent on the number of cells with a normal karyotype identified, guaranteeing a better representation of the population under study.

### 2.8. Immunohistochemical Analysis

Early passage (P5) of OM-MSCs was submitted to immunohistochemical analysis to detect specific antigens (Table 1). Cells were maintained in culture until a confluence of 70–80% was reached, then enzymatic detachment was performed with 0.25% Trypsin-EDTA solution and a paraffin cytoblock (SureThin® Preserve Cell solution®, Cytoglobe GmbH®) was accomplished. Sections were cut at 2 *μ*m, deparaffinized, dehydrated and submitted to immunohistochemical analysis using the Novolink™ Polymer Detection Systems (Leica Biosystems®) kit, according to the manufacturer's instructions. Information regarding the primary antibodies and antigen retrieval methods used in the study are summarized in Table 1. The antibodies were selected to confirm the mesenchymal origin of OM-MSCs (vimentin and c-Kit), to discard an endothelial (CD31) and epithelial (Cytokeratin AE1/AE3) origin and to identify the expression of neural (synaptophysin) and glial markers (GFAP).

The samples were observed, evaluated, and photographed using the microscope Eclipse E600 (Nikon®) and the software Imaging Software NIS-Elements F Ver4.30.01 (Laboratory Imaging®). Immunoexpression of the different markers was scored for the percentage of labeled cells (<5%; 5–80%; >80%) and labeling intensity (0, negative; +, weak; ++, moderate; +++, strong). Immunoreactivity was considered as positive when distinct nuclear and cytoplasmatic staining was recognized in at least 5% of the cells.

### 2.9. OM-MSCs' Conditioned Medium Analysis – Secretome

To identify specific chemokines and growth factors produced and secreted by OM-MSCs, conditioned medium (CM) was analyzed. For the conditioning, OM-MSCs in early passage (P4) were applied. Once 70–80% confluence was reached, the culture medium was removed, and the culture flask gently washed with PBS 5 times. The culture flask was further washed twice with culture medium. Subsequently, basal culture medium without supplementation with antibiotic, antimycotic, or FBS was added to the culture flask, which was incubated under standard conditions for 48 h. After this period, the culture medium rich in factors secreted by the cells (conditioned media) was collected, centrifuged, and the supernatant collected. CM was stored at −20°C and subsequently analyzed by Multiplexing LASER Bead analysis (Eve Technologies, Canada) to search for a specific set of biomarkers (TM-Featured Cytokine Array/Chemokine Array 8-plex (RECYT-08-204)). The biomarkers studied were Interferon gamma (IFN-*γ*), monocyte chemotactic protein-1 (MCP-1), tumor necrosis factor alpha (TNF-*α*), vascular endothelial growth factor (VEGF) and several interleukins (IL-2, IL-4, IL-6, IL-18). Six independent samples of CM were analyzed.

### 2.10. Statistical Analysis

Statistical analysis was performed using the software GraphPad Prism version 6.00 for Windows (GraphPad Software, La Jolla California USA). When appropriate, data were expressed as mean ± SEM. Comparisons between groups were performed with an analysis through a parametric test. A value of *P* < 0.05 is considered statistically significant. Significance of the results is showed according to *P* values by the symbol (∗), (∗) corresponding to 0.01 ≤*P* <0.05, (∗∗) to 0.001 ≤ *P* < 0.01, (∗∗∗) to 0.0001 ≤ *P* < 0.001 and (∗∗∗∗) to *P* < 0.0001.

## 3. Results

### 3.1. Isolation and *In Vitro* Expansion of OM-MSCs

**Figure 1:**
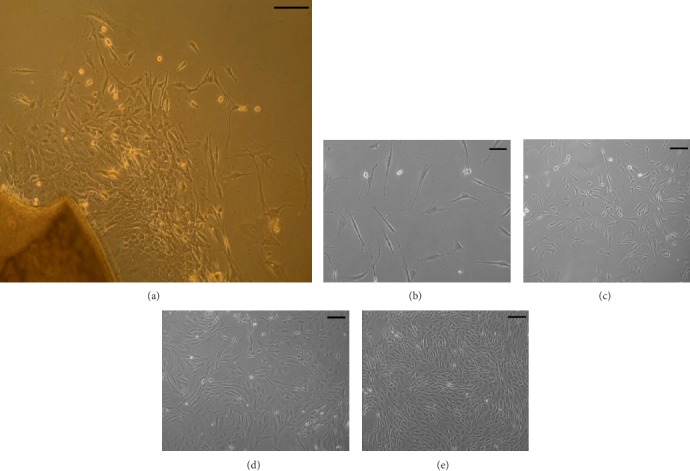
Magnification: 100x; Scale Bar: 100 *μ*m. (a) OM fragment plated by the explant method after enzymatic digestion. 1 week after plating, cells with morphological characteristics of MSCs irradiating from the explant can be observed; (b)–(e): morphology of OM-MSCs in culture: (b) P2, 30–40% confluence; (c) P5, 50–60% confluence; (d) P7, 70–80% confluence, ideal moment for passage; (e) P9, 90–100% confluence.

The procedure performed to isolate OM-MSCs from OM is mixed, comprising explant plating after an enzymatic treatment. This method showed efficacy and allowed observation of OM-MSCs radiating from the explants and harvesting was performed within 1 to 2 weeks after plating of the explant (Figure 1(a)).

### 3.2. Cell Behavior in Culture

Both cells observed radiating from the explants and those identified in culture showed clear plastic adhesion and mostly fibroblast-like morphology, an essential feature to characterize cells as MSCs (Figures 1(b)–1(e)). Considering earlier passages, adhesion time was longer and cell growth and proliferation slower. Cell performance, as for adhesion and proliferation, was maximal between P5 and P8. At this phase, lower time for adherence, unequivocal fibroblast-like shape, and shorter time to reach desired confluence was observed. Performance decreased progressively after passages P8-P9.

#### 3.2.1. Growth Kinetics and Cell Viability

**Figure 2:**
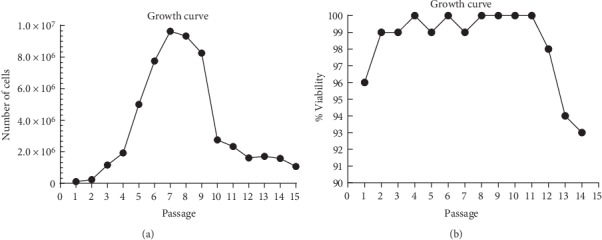
(a) OM-MSCs' growth curve over 35 days (15 passages); (b) Variation of % cell viability over 35 days (15 passages).

The growth curve was achieved by analyzing the OM-MSCs over 15 passages (P15). The number of cells quantified remained stable in the first two passages, with an exponential increase up to the seventh passage. After this phase, a marked decrease in the number of cells quantified was observed, and this value remained stable until P15 (Figure 2(a)). Cell viability was lower in the first passage. Between passage 2 and passage 12% viability remained between 99% and 100%. In the last three passages the viability decreases progressively (Figure 2(b)).

#### 3.2.2. PDT

**Figure 3:**
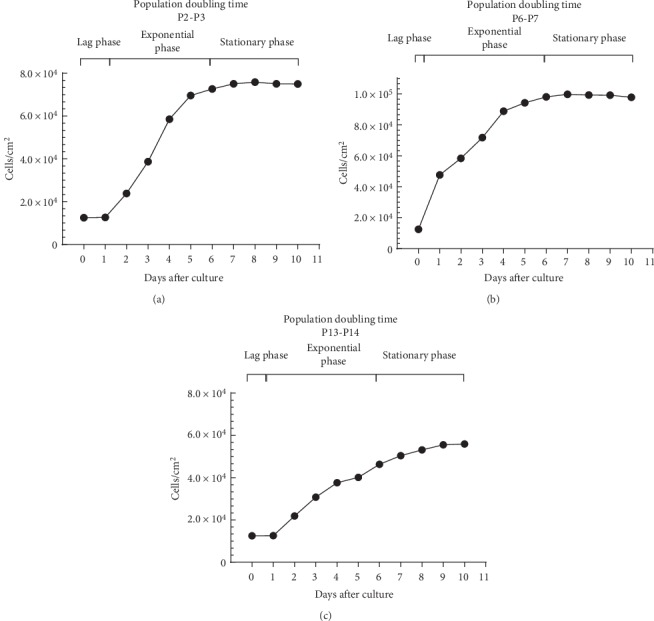
Cell growth of OM-MSCs over 10 days of culture in P2-P3 (a), P6-P7 (b), and P13-P14 (c).

**Figure 4:**
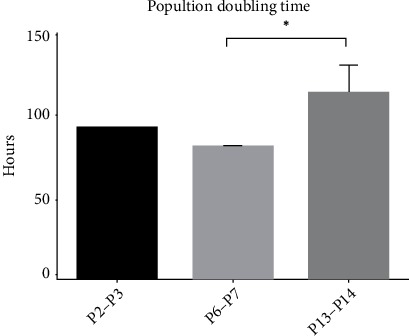
PDT for the different passages (mean ± SEM).

The growth curve for cells growing in culture had an identical shape for the three determinations of PDT with respect to the exponential and stationary phases (Figure 3). Regarding the *lag* phase, it was easily identifiable in passages P2-P3 and P13-P14, being not visible in the graph P6-P7. The mean value of PDT was 92.87 ± 0.04 hours for the passage P2–P3, 80.94 ± 0.79 hours for P6-P7 and 113.99 ± 18.01 hours for P13-P14 (Figure 4).

#### 3.2.3. Colony Forming Unit Assays

**Figure 5:**
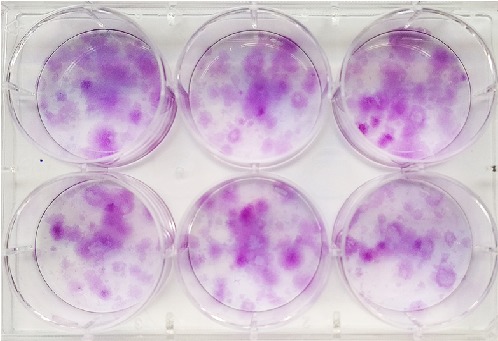
CFU assay: observation of individualized colonies in a 6-well tissue culture treated plate, stained with crystal violet solution.

CFU assays allowed to confirm the ability of OM-MSCs to generate new fibroblast colonies from single cells. After 14 days of incubation, it was possible to identify an average of 35.33 ± 3.56 colonies with spindle morphology per well, approximately 212 colonies up to 1 mm formed from around 480 seeded cells. The % of clonogenicity was 44.16 ± 4.45% (Figure 5).

#### 3.2.4. Cryopreservation and Thawing

OM-MSCs submitted to cryopreservation and thawing procedures showed no detectable performance changes. Independently of the passage in which the cryopreservation occurred, after thawing, cell adhesion to the plastic surface occurred in few hours, confluence of 70–80% was reached between 3 and 4 days and the expected morphology for OM-MSCs (fibroblast-like shape) was also observed. Thawing of cells below P5 (*N* = 12), the % cell viability determined by Trypan blue exclusion cell assay was 97.83 ± 1.07%, with viability above 95% for all the samples tested.

### 3.3. Differentiation Protocols

#### 3.3.1. Adipogenic Differentiation and Oil Red O Staining

**Figure 6:**
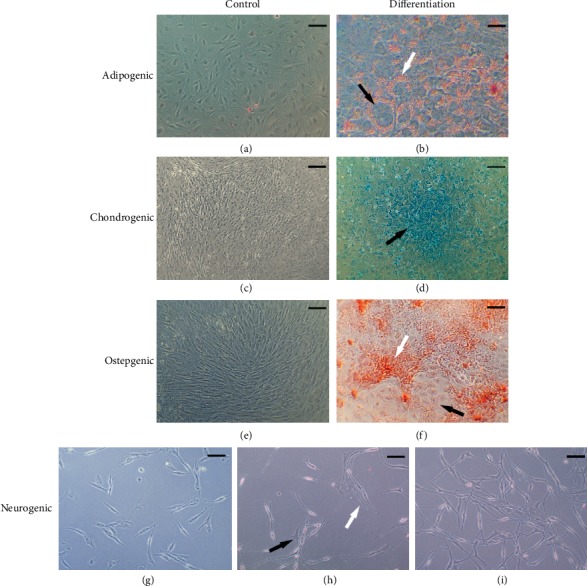
Cell differentiations. Magnification: 100x; Scale bar: 100 *μ*m. Adipogenic differentiation: (a) control; (b) presence of large round shaped cells (black arrow) and red stained lipid vacuoles inside the cytoplasm (white arrow); chondrogenic differentiation: (c) control; (d) presence of chondrogenic aggregate and extracellular matrix stained in blue (black arrow); osteogenic differentiation: (e) control; (f) presence of calcium containing osteocytes (black arrow) and extracellular calcium deposits (white arrow) stained in red; neurogenic differentiation: (g) control; (h) 24 h after addition of neurogenic induction medium, the cells acquired a neuroglial-like shape with development of dendrite (black arrow) and axonal-like (white arrow) cell structures (i) 72 h after addition of neurogenic induction medium.

The ability of OM-MSCS for adipogenic differentiation was confirmed by observation of morphologic changes (large cells with a rounded shape) and red stained lipid vacuoles in the cytoplasm due to exposure to Oil Red O solution. (Figures 6(a) and 6(b)).

#### 3.3.2. Chondrogenic Differentiation and Alcian Blue Staining

The ability of OM-MSCS for chondrogenic differentiation was confirmed by observation of chondrogenic aggregates and extracellular matrix stained blue due to the exposure of the proteoglycans to Alcian Blue solution (Figures 6(c) and 6(d)).

#### 3.3.3. Osteogenic Differentiation and Alizarin Red Staining

The ability of OM-MSCS for osteogenic differentiation capacity was confirmed by identification of calcium containing osteocytes and extracellular calcium deposits red stained due to the exposure to Alizarin Red solution (Figures 6(e) and 6(f)).

#### 3.3.4. Neurogenic Differentiation

On day 1 after replacement of the culture medium by neurogenic induction medium, clear morphological alterations were already observed in the OM-MSCs that acquired a neuroglial-like shape with development of axon and dendrite-like cell structures (Figures 6(g)–6(i)). The neuroglial-like morphology was maintained throughout the 5 days of observation, but an increase in the mortality rate and number of cell debris in suspension were also identified.

### 3.4. RT-PCR

**Figure 7:**
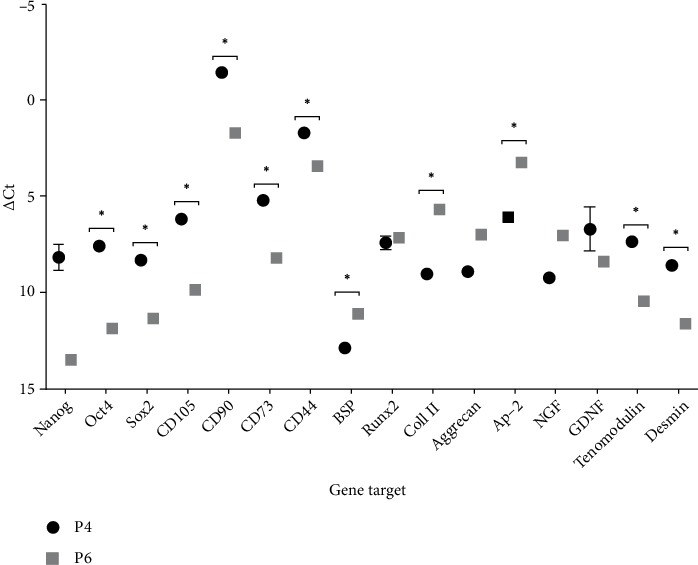
ΔCt Values for the different genes under study in OM-MSCs for P4 and P6. Higher delta-CT values represent lower expression (mean ± SEM).

Table 2 shows the Ct average values for each gene, ΔCt, ΔΔCt and RQ values. According to the analysis performed, both samples showed purity, allowing their use in subsequent phases. Cells at P4, CD90 and CD44 showed Ct values <29. All other genes, except for BSP, had average Ct values between 30-39, indicating positive reactions and moderate amounts of target nucleic acid in the sample. Cells at P6, once again CD90 and CD44, and also Ap-2 showed Ct < 29, that is, abundance of the target nucleic acid in the sample, and all other genes presented values between 30 and 39. In both passages, CD45, CD34 and AdipoQ genes had no detectable expression. Statistically significant differences were identified between ΔCt values of the genes Oct4, Sox2, CD105, CD90, CD73, CD44, BSP, Coll II, Ap-2, Tenomodulin an Desmin for P4 and P6 (Figure 7). Comparing the two passages, changes were observed in fold-expression of the genes. At P6, NANOG, OCT4, Sox2, CD105, CD90, CD73, CD44, GDNF, Tenomodulin and Demin appear down-regulated and Ap-2, BSP, Coll II, aggrecan and NGF appear up-regulated.

### 3.5. Cytogenetic Analysis

**Figure 8:**
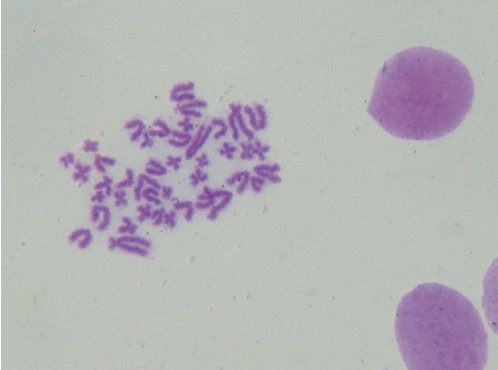
OM-MSCs' karyotype (P8), showing structural normality, normal number of chromosomes (42, XY) and absence of neoplastic alterations. These characteristics demonstrate the chromosomal stability of these cells.

Cytogenetic evaluation allowed the identification of a high percentage of cells with a normal karyotype for the species (42, XY) for all passages (Figure 8), although a small decrease in this percentage was observed in passages P8 and P11 compared to passage P5. Regarding the mitotic index, the values are normal for passages P5 and P8 and low for passage P11 (Table 3).

### 3.6. Immunohistochemical Analysis

**Figure 9:**
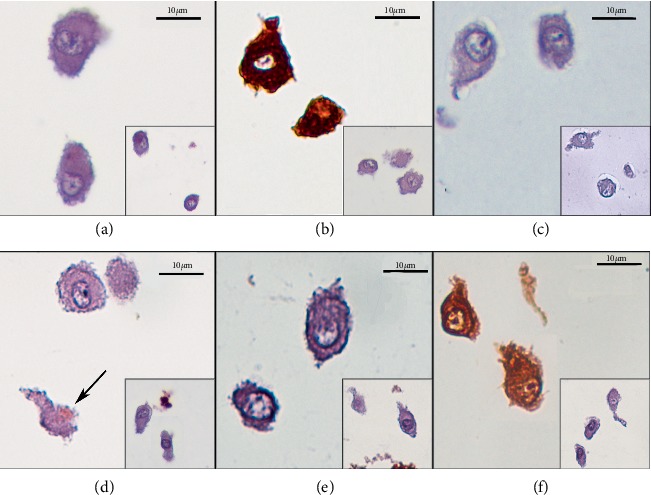
Immunolabeling of rat OM-MSCs (P5), Magnification: 600x: (a) AE1/AE3 (0); (b) Vimentin (+++); (c) CD31 (0); (d) Synaptophysin (+), (the black arrow highlights focal and membranous positive immunostaining); (e) c-Kit (++); (f); GFAP (+++). Insets show the respective negative controls for each immunomarker.

Immunohistochemistry, OM-MSCs showed weak immunoreactivity for synaptophysin (comprising about 5% of cell population), moderate for c-kit (comprising 5–80%) and strong staining for GFAP and Vimentin (involving >80% of the cells). Cells were negative for AE1/AE3 and CD31 immunomarkers (Figure 9).

### 3.7. OM-MSCs' Conditioned Medium Analysis – Secretome

**Figure 10:**
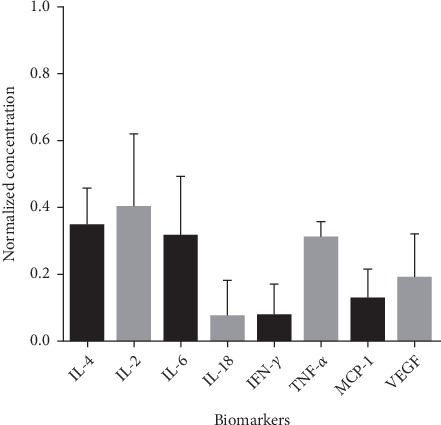
Normalized concentration of each biomarker in the conditioned medium of OM-MSCs (mean ± SEM).

The mean concentration of each biomarker in the CM under analysis is shown in Table 4 and Figure 10.

## 4. Discussion

Even though multipotent cells in the OM has been known for a long time [17], only recently the OM-MSCs have been adequately identified. Efforts have been made to characterize these cells in different species, and to establish appropriate collection, isolation, characterization, storage, and experimental application methods. In the present work, an extensive characterization of rat OM-MSCs was carried out to confirm their characteristics of stem/stromal cells and their potential use in future regenerative therapies. The authors opted for a detailed description of all the procedures performed, since variations in manufacturing processes can lead to difficulties in comparing the results obtained in different laboratories and in different works, and many of these differences can be explained through the variations in the established protocols [11].

Although the niche of OM-MSCs is helpful because of their peripheral position in the body, the remote location of the olfactory recess and OM within the nasal cavity and the intricate anatomic characteristics of the nasal turbinates that make access to this area, significantly hamper the OM tissue collection [51]. The protocol applied in this work is a fast and efficient alternative for collection of OM fragments from the rat's nasal cavity, with no need for complex procedures or specific surgical instruments. Care should be taken to avoid an overly aggressive approach to the nasal cavity that may trigger hemorrhage and contamination of OM. [24] Solutions for transporting and storing fragments of OM before processing are also important for tissue preservation and integrity of OM-MSCs. Here, as in all procedures, we chose to use PBS because of its ability to prevent cells rupturing or shriveling up due to osmosis [52]. Antibiotics and antimycotics were used because of collection procedure of OM and its anatomical location, prone to contamination, as to avoid bacterial or fungal contaminations.

Aseptic conditions were maintained when performing all procedures of isolation and culture. For cell culture, besides the antibiotic and antimycotic use already mentioned, the culture medium comprised a basal medium rich in glucose, amino acids, and vitamins, and FBS for animal-derived growth supplementation. All culture media were sterile filtered (Acrodisc® Syringe Filters with Supor® Membrane, 0.2 *μ*m), and cell culture incubations was performed under standard conditions (37°C, 5% CO_2_ humidified atmosphere). All these manufacturing practices ensured the maintenance of healthy and functional cell cultures with no contamination throughout the study period and in all the procedures performed.

Mixed isolation of OM-MSCs from OM fragments was effective. In the period between 1 and 2 weeks after plating of explants, it was possible to observe irradiant spindle-shaped cells in the plate, as detected in other works performed with rat [22] and other species [34, 46]. Although some authors prefer a simple method without performing enzymatic digestion of the OM fragments [28, 53], the enzymatic treatment before plating the explants did not affect the characteristics of OM-MSCs and was corroborated by other authors [30]. After isolation of OM-MSCs and their culture, it was possible to observe their features of MSCs [10], namely plastic adhesion and fibroblastic-like shape, as confirmed by other authors [29, 54]. For higher passages, morphological alterations could be observed, with the cells appearing more rounded and losing the characteristic elongated shape, some of them detaching, and increasing the amount of cellular debris suspended in the medium. These changes associated with a high number of passages are common and characteristic of MSCs in long-term cultures [55].

The growth kinetics can be studied through the growth curve and the PDT, concepts that should not be considered as synonymous. While the growth curve allows comparing the number and characteristics of cells at the same degree of confluence along the different passages, with the number of cells being determined only at the time of the passage itself, PDT shows the time it takes for the number of cells in culture to increase to double and is obtained by daily determinations of cell numbers along the same passage. Through the determination of PDT it is also possible to plot growth curves over the days of the same passage. While the two studies can be done together as in the present work, PDT is a better indicator of cell performance since cell growth can vary greatly between donors and methods of preparation. PDT correlates directly with genetic stability and with replicative senescence, linked to loss of potency. In addition, PDT makes it possible to analyze and compare different studies easily [56]. The growth curve observed is identical to that described for OM-MSCs from other species [26]. Growth and cell viability curves showed poorer cell vitality in the lower and higher passages, in which a lower number of cells in the passage and lower cell viability were observed. Cell viability should be at least 95% for healthy cultures [57] and from P13 values lower than these were observed. Growth curves analyzed for PDT determination confirmed these observations. The growth of MSCs must occur in 3 phases: initial lag phase, fast and exponential growth phase, and stationary phase [58]. These three phases are present in the growth curves of OM-MSCS at the 3 moments considered. Finally, PDT is lower for P7–P8, followed by P2–P3, and passage P13–P14 has the worst result. Recalling, the lower the PDT, the faster an increase to twice in cell efficiency. The PDT values observed in this work are higher than those described for rat's OM-MSCs in other works [29]. All these findings, together with the observed behavior of OM-MSCs in culture, confirmed the occurrence of senescence phenomena during extended periods culture [59]. Senescence phenomena start when MSCs are cultured and progressively worsens over time and along passages, with gradual decreasing of proliferation potential, telomere shortening and impairment of functions [55, 60]. Morphologically the senescence manifests through debris in the medium and granules in the cytoplasm of the cells [61]. All of these phenomena were observed in OM-MSCs under study, even though earlier studies have reported self-renewal in long-term cultures of olfactory neuro-epithelial derived progenitors by maintenance of telomerase activity and lack of apoptotic activity [23]. Thus, rat's OM-MSCs should be assigned to cell therapies ideally in early stages of *in vitro* culture, ideally between P4 and P8.

The determination of CFUs is a good method to assess self-renewal efficiency and the ability of MSCs to originate colonies from a single precursor cell [62]. The number of colonies formed from a specific number of seeded cells represents the colony-forming efficiency and can be defined as the clonogenicity of the cell population. In the present study, as in other works focusing on OM-MSCs [15, 26], cells originated several colonies with spindle morphology, presenting a clonogenicity with values close to those found in the literature for the specie [29].

Cryopreservation is an important method to preserve MSCs, allowing almost immediate availability of cells for clinical application in cell therapies and regenerative medicine. During the cryopreservation and subsequent thawing process, it is important that MSCs maintain their intrinsic properties, namely their culture behavior, their immunomodulatory characteristics, and the ability to multilineage differentiation [63]. The changes and effects caused by the different methods of cryopreservation, medium, cryoprotectants, temperature, and duration of cryopreservation are relatively well defined [64], but there is little clear information in this subject regarding MSCs of veterinary origin. The choice of the cryopreserving agent is an important aspect. In the present work we opted for the use of DMSO due to the evidence that this agent guarantees greater cell viabilities after the cryopreservation and thawing cycle. Its mechanism of action during cryopreservation is based on its ability to penetrate cells and remove water from them, thus preventing intracellular ice formation and cell rupture [65]. Some studies indicate that DMSO has tumorigenic potential when in contact with cells, but the possible similar effect of other cryoprotective agents has not yet been adequately established [66, 67]. Other works also indicate that DMSO above 4°C can become cytotoxic [66]. To avoid this effect, during the thawing phase it is ensured that there is no complete thawing of the ice within the cryovial, that the cryopreservation solution is readily centrifuged, and the supernatant containing the DMSO eliminated. The duration of maintenance of MSCs in cryopreservation may also affect their characteristics [68]. Cells used in this work did not exceed twelve months of cryopreservation, so it is not possible to anticipate possible negative effects from longer storage periods. In the procedures performed using the described protocol of cryopreservation and thawing, no specific alterations were observed regarding cell adherence after plating, cell confluence, number of cells and viability during cell passage.

Although multipotent capacity of MSCs is widely established in several species and in different niches [11], the capacity for tridifferentiation continues to be used as a minimum criterion for identification and characterization of these stem/stromal cells [10]. In the present study, after the establishment of specific differentiation protocols, OM-MSCs showed a capacity for adipogenic, osteogenic, and chondrogenic differentiation in culture. The capacity for differentiation into a neuroglial-like lineage was also tested and confirmed, although further studies are needed to determine the actual efficacy for neurogenic differentiation. The early transformation of OM-MSCs into a neuroglial-like lineage following exposure to neurogenic differentiation medium could also be observed, but it is important to note that simple morphological differentiation is not sufficient to consider the functional efficacy of neurogenic differentiation and further studies are needed to determine this effectiveness. These capabilities confirm the results obtained by other studies focusing on OM-MSCs [15, 26, 28, 29, 32, 69].

RT-PCR allowed to quantify the gene expression of 19 genes associated with OM-specific markers on OM-MSCs and identify changes in their fold-expression between passages. The determination of the purity by spectrophotometry allowed to confirm the absence of contamination by different origins in the RNA samples used to get cDNA. Likewise, the study of melting curves and the observation of a single peak in each of them also allowed to confirm the amplification of a single amplicon for each gene under study. Of the 19 genes under study, no genetic expression was observed for 3 of them in both passages: CD34, CD45 and AdipoQ. The absence of CD34 and CD45 markers is considered a minimum criteria for the characterization of cells as MSCs [15]. The absence of CD34 excludes a primitive hematopoietic origin [70] and the absence of CD45 excludes a leukocyte origin [71]. AdipoQ is an adipogenic gene [72]. Considering the previously mentioned ability of OM-MSCs for adipogenic differentiation, the nonexpression of this gene is unexpected. Both in P4 and P6 some genes presented a Ct < 29, that is, abundant target nucleic acid in the sample. CD90 is used as a minimum criterion for MSCs' classification [15], being related to cell repair and cell to cell interactions phenomena [11]. Its high genetic expression is expected and confirmed by other studies of OM-MSCs [26]. CD44 is used as a specific marker for classifying human OM-MSCs [73], although the classification for veterinary cells does not always consider this marker [11]. Since CD44 is closely related to cell adhesion phenomena [74], its high expression at OM-MSCs is also expected. AP-2 gene, related to the capacity of adipogenic differentiation [75] also shows high expression in P6, although with a lower value in P4. CD73 and CD105, respectively related to adenosine production [76] and vascular hemostasis phenomena [77], are also considered as minimum criteria for classification of OM-MSCs. In both passages their Ct values were lower than 35, so that their genetic expression can be considered moderately high. Still with Ct values below 35 are several genes associated with specific markers of osteogenic (Runx2 in P6) [78], chondrogenic (Coll II in P6, Aggrecan in P6) [79], adipogenic (AP-2 in P4) [75], and neurogenic (NGF in P6, GDNF in P4 and P6) [80, 81] differentiations, confirming the ability to tridifferentiation and the potential for differentiation in mesodermal lines [29]. Even with higher Ct values, other indicators of tenogenic differentiation (Tenomodulin in P4 and P6,) [82] osteogenic differentiation (BSP in P6) [83] and musclogenic differentiation (Desmin in P4 and P6) [84] also presented relevant gene expression. More irregular is the value of Ct greater than 35 for genes related to multipotency capacity such as NANOG, Oct4 and Sox2 in both passages. These markers are considered regulators of pluripotency of MSCs [85], although some studies raise doubts as to the influence of all these markers in this multipotentiality [86]. These genes are also down-regulated at P6 compared to P4, indicating a possible loss of propensity for pluripotential activity the cell population at higher passages. In contrast, most of the differentiation-related genes are up-regulated at P6, which may be explained by an increase in the propensity of these cells for specific differentiations in the interval for which the maximum functional activity of OM-MSCs was established (P5-P8). This suggests that these cells, although with greater up-regulation of genes associated with pluripotency at earlier stages, at around passage 6, present a greater propensity for specific differentiations, possibly being this the ideal moment for clinical application. These results should be interpreted with caution. The minimum criteria used to characterize human MSCs are also applied to MSCs of animal origin, even though several studies have shown that there is no precise parallelism in the expression of these surface markers [29, 87–91]. In addition, in-depth studies are needed to identify markers that can be considered as unique and unambiguous in the characterization of veterinary MSCs [92]. However, considering current knowledge, RT-PCR corroborates the mesenchymal origin of the rat's OM-MSCs and their tendency for multidifferentiations.

The cytogenetic evaluation of OM-MSCs allowed the identification of chromosomal stability, with a high percentage of normal karyotypes at all passages. This percentage, however, is lower in higher passages. Similarly, whereas in passages P5 and P8 the mitotic index (i.e., the number of cells in mitosis in relation to the total number of cells analyzed) is normal, for passage P11 it decreased. In all passages the cells were analyzed after reaching a confluence of 70–80%, a value that was observed more quickly in the lower passages. This means that in passage P11 a lower mitotic activity is observed for the same level of confluence. The lower mitotic index for passage P11 corroborates the results observed in the remaining tests, where some morphological alterations, poorer cellular vitality, a lower number of cells in the stationary phase and lower cell viability were observed for higher passages, indicative of senescence phenomena.

Vimentin is a widely expressed and highly conserved 57-kD protein that constitutes type III intermediate filaments and is constitutively expressed in mesenchymal cells, being important in their development, integrity, and maintenance [93], and modifications in these intermediate filaments can lead to morphological and functional changes [94, 95]. Vimentin is often identified on the surface and intracellular filament of MSCs and different tissues, namely the OM [96] and on olfactory receptor neurons which continue to express this marker unlike other postmitotic neurons that cease vimentin expression as nerve development progresses [97]. Having been previously identified in mice OM-MSCs [45] and considering its expression in cells derived from the neural crest [98], vimentin can therefore be used as a MSC marker on the OM. In addition, c-Kit is a type-III receptor tyrosine kinase that transduces cell signaling events by binding to its ligand, stem cell factor, and regulates cell proliferation, differentiation, chemotaxis, adhesion, and apoptosis [99]. Thus, it is possible to identify c-Kit immunoexpression in cell populations of mesenchymal origin [100] and MSCs [101, 102], and this marker is also expressed in the embryonic olfactory mucosa and olfactory epithelial progenitor cells, which are necessary for the maintenance of this neuroepithelium and which contribute to olfactory neurogenesis [103]. As such, both vimentin and c-Kit can be considered as useful markers for MSC identification on the OM, and their detection in this current OM-MSCs isolated population corroborates its histogenesis and further classification. About 5% of OM-MSCs stained positively for synaptophysin and >80% strongly labeled with GFAP. Synaptophysin is an antigen related to the synaptic vesicles of neuroendocrine tissues, appearing in the rat's olfactory system at OM's olfactory neurons [96] and also in olfactory bulb's glomeruli [104]. GFAP is a fibrillary protein characteristic of glial cells and whose expression is characteristic in cells of the nervous system [105]. GFAP was already identified in OM-MSCs [19] and in MSCs subject to neurogenic differentiation [106, 107]. The spontaneous expression of neural markers by MSCs in their undifferentiated state has already been showed for different cell types, and is considered by some authors as an evidence of the MSC predisposition to differentiate into a neurogenic line [108]. Positive staining of undifferentiated OM-MSCs with synaptophysin and GFAP evidences the propensity of these cells for neurogenic differentiation, also herein verified with the neurogenic differentiation protocol. It is important to note that, a higher percentage of GFAP-labeled cells was identified in OM-MSCs when compared with other undifferentiated MSCs, at identical passage [108]. This might be related to the niche where the OM-MSCs were collected since the OM is naturally rich in neuroregenerative activity, which may turn these cells more predisposed for neurogenic differentiation. CD31 is an endothelial adhesion molecule associated with endothelial and hematopoietic cells, thus its absence of expression in OM-MSCs is expected [109]. AE1/AE3 are cytokeratins related to the cytoskeleton present in almost all epithelia and are also useful for identifying and differentiating epithelial tumors [110]. Lack of expression of both CD31 and AE1/AE3 markers eliminates an endothelial and epithelial nature respectively, for OM-MSCs. In summary, immunohistochemical analysis reinforced the mesenchymal origin of OM-MSCs and confirms the tendency of these cells for neural differentiation.

The CM of MSCs is the set of substances extracted from the medium in which the MSCs are cultured and contain a high number of soluble factors such as cytokines, chemokines, immunomodulatory molecules, and growth factors with potential regenerative effect [111, 112]. The majority of these secreted factors found in MSC derived cell-free secretome demonstrate properties and effects identical to those described in the MSCs themselves, related to processes of intercellular communication, cell signaling, differentiation, and cell adhesion, and include elements such as extra and intracellular matrix proteins, inflammation-related cytokines, chemokines, and angiogenic factors [113]. The proregenerative effects of MSC secretoma have been observed in several tissues and involve actions of modulation of the immune system, inhibition of cell death and fibrosis, stimulation of vascularization, promotion of tissue remodeling, and cell recruitment [114]. In the present study, several biomarkers were investigated and their concentration in the CM after 48 h of OM-MSCs conditioning was measured. The biomarkers under study are grouped into immunomodulatory and immunosuppressive factors (IFN-*γ* and TNF-*α*), chemokines (MCP-1), growth factors (VGEF), and interleukins (IL). All biomarkers were identified in the CM although with different concentrations. The relatively low concentrations of some of these biomarkers should not be surprising and does not necessarily affect their therapeutic efficacy when applied *in vivo*, since the therapeutic concentration of these factors in a delivery system generally lie in significant low ranges [115]. The concentration of INF-*γ* in the CM is relatively low, and TNF-*α* values are higher. At an organic level, the presence of these factors, as well as the presence of infectious toxins or hypoxia environment, stimulate the activation of MSCs and the production of specific growth factors and chemokines [116]. In fact, the combination of IFN-*γ* and proinflammatory cytokines such as TNF-*α* not only stimulates MSCs to produce and secrete high levels of immunosuppressive factors, as well as chemokines, but also adhesion molecule expression and vascular cell adhesion molecule expression [117]. The cell conditioning applied over 48 h seems to have been enough to stimulate the release of factors not generally produced by MSCs such as TNF-*α* and IFN-*γ*, which in turn stimulated the OM-MSCs to produce growth factors (VEGF) and chemokines (MCP-1). VEGF, besides its functions as an endothelial growth factor, plays important roles in the survival of MSCs in extreme organic conditions [118, 119]. The environmental conditions experienced by cells during conditioning may also be an important element in the production and secretion of provasculogenic factors such as VEGF by MSCs [120]. MCP-1, in turn, acts as a potent chemoattractant that stimulates the recruitment and proliferation of MSCs and fibroblasts [121], and its presence in the secretome has already been reported in other OM-MSCs [113]. Taken together, the production of chemokines and growth factors by OM-MSCs subjected to conditioning has as main objective to stimulate cell recruitment, migration and to guarantee cell proliferation and survival under extreme environmental conditions caused by the conditioning process. IL-6 is secreted by MSCs and was initially thought to play proinflammatory functions. Recently, its suppressive action in the activity of T cells and in the control of local inflammation has been identified, thus performing an immunoregulatory function [122, 123]. Therefore, IL-6 plays an immunosuppressive action and may play an important role in avoiding organic rejection during allogeneic implantation of MSCs [124]. The production of this interleukin by OM-MSCs can be an indicator of the safety on the use of these cells in a therapeutic way, because of its immunosuppressive effect and help in avoiding the organic rejections to the implanted cells. IL-4 plays an important anti-inflammatory role and has an inhibitory effect on the production of TNF-*α* [125], which may explain the similar concentrations of these two factors. IL-18 is a proinflammatory cytokine related to the induction of inflammation and cell death and is thought to have inhibitory effects on cell proliferation and apoptosis phenomena [126, 127]. Despite this, some studies have shown the beneficial effects of these interleukins, for example, on suppressing breast cancer cells *in vitro *[128] and intracranial gliomas [129], playing an antitumor effect by stimulating the activity of natural killer cells, reducing tumorigenesis, inducing tumor cell apoptosis, and inhibiting tumor angiogenesis [130]. Regardless the clarification of its effects, the identification of this interleukins in CM evidences its involvement in the immunomodulatory response of MSCs. Unexpected is the relatively high concentrations of IL-2 in the CM of OM-MSCs. MSCs can control the production and secretion of IL-2 and *T* cells IL-2 response through the secretion of IL-10 [131, 132]. It is possible that the hostile environment triggered by the conditioning of OM-MSCs over 48 h may be enough to induce a proinflammatory response and production of IL-2, which would also explain the production and secretion, although to a lesser extent, of other biomarkers such as TNF-*α*, IFN-*γ* and IL-18. Despite the several studies already carried out, there seems to be no consensus regarding the components that must be found in the MSC secretome [133], which can be affected by aspects such as the assay used to obtain the CM, the niche of the MSCs, the conditioning period, and the number of passages of the cells used to obtain it [134]. Since OM-MSCs are sensitive to the environment in which they are found and alter their secretion profile according to signaling factors and surrounding environment, changes in some cells within the culture can rapidly trigger and stimulate neighboring cells to produce and secrete the same factors, greatly altering the CM and the secretome in question [133]. The major immunomodulatory agents are the inflammatory cytokines [135], growth factors used as a supplement in the culture medium [136], and also the reduced oxygen tension [137].

## 5. Conclusion and Further Directions

OM-MSCs have recently become a promise in regenerative medicine because of their peripheral location that makes them easy to collect, isolate, and expand in culture and because of their biological characteristics already established. With the methodologies applied in the present work it was possible to establish a protocol for isolating, culturing, freezing, and thawing the OM-MSCs without altering their morphological or behavioral characteristics in culture. Sequentially, a biological characterization was performed, allowing to identify a normal karyotype, several genes characteristic of MSCs and differentiations, ability for tridifferentiation and neurogenic differentiation and specific surface markers. The stemness characteristics of the OM-MSCs were also recognized, allowing to establish these cells as adequate to be applied therapeutically in future works.

Cell therapies are established as a therapeutic promise, but different obstacles related to their safe use, standardization of processes, and mechanisms of cell delivery into injured tissues are promoting increased attention to the therapeutic effects of MSCS' paracrine secretory factors. The study of the OM-MSCs' CM allowed the identification and quantification of different biomarkers related to immunomodulation capacity, creating the possibility of using the secretome of these cells in future regenerative therapies.

## Figures and Tables

**Table 1:** List of antibodies investigated, dilutions and antigen retrieval methods applied in the immunohistochemical analysis.

Antibody	Clonality	Manufacturer	Dilution	Antigen retrieval
Cytokeratin	Clone AE1/AE3	Invitrogen®, Thermo Fisher Scientific®	1/200	Water bath/20 min
Vimentin	Clone V9	DAKO®, Agilent Technologies®	1/1200	Water bath/20 min
CD31	Clone JC70A	DAKO®, Agilent Technologies®	1/200	Pepsine
Synaptophysin	Clone SP11	Thermo Fisher Scientific®	1/100	Water bath/20 min
c-Kit	Polyclonal	DAKO®, Agilent Technologies®	1/450	Water bath/20 min
GFAP	Polyclonal	Millipore®	1/2000	Water bath/20 min

**Table 2:** Ct, ΔCt, ΔΔCt and RQ values for all genes under study for P4 and P6. nd = nondefined.

Target gene	P4		P6				
	Ct average OM-MSCs	ΔCt OM-MSCs	Ct average OM-MSCs	ΔCt OM-MSCs	ΔΔCt	RQ	Regulation
Nanog	35.9 ± 0.9	9.7	38.2 ± 0.0	13.5	5.4	0.0	↓
Oct4	35.3 ± 0.0	9.1	36.5 ± 0.0	11.9	4.3	0.1	↓
Sox2	36.0 ± 0.0	9.8	36.0 ± 0.0	11.3	3.0	0.1	↓
CD105	33.9 ± 0.0	7.7	34.5 ± 0.0	9.8	3.7	0.1	↓
CD90	26.2 ± 0.2	−1.5	26.4 ± 0.0	1.7	3.2	0.1	↓
CD73	32.9 ± 0.3	6.7	32.9 ± 0.3	8.2	3.0	0.1	↓
CD44	29.4 ± 0.1	3.2	28.1 ± 0.1	3.4	1.7	0.3	↓
CD45	nd	nd	nd	nd	nd	nd	nd
CD34	nd	nd	nd	nd	nd	nd	nd
BSP	40.6 ± 0.0	14.4	35.8 ± 0.0	11.1	−1.8	3.5	↑
Runx2	35.1 ± 0.6	8.9	31.8 ± 0.0	7.1	−0.3	1.2	N
Coll II	36.8 ± 0.0	10.6	30.3 ± 0.3	5.7	−3.4	10.5	↑
Aggrecan	36.6 ± 0.6	10.4	31.6 ± 0.4	7.0	−1.9	3.8	↑
Ap−2	33.8 ± 0.4	7.6	27.8 ± 0.1	3.2	−2.9	7.5	↑
AdipoQ	nd	nd	nd	nd	nd	nd	nd
NGF	36.9 ± 0.7	10.7	31.7 ± 0.4	7.0	−2.2	4.5	↑
GDNF	34.4 ± 1.7	8.2	33.0 ± 0.0	8.4	1.7	0.3	↓
Tenomodulin	35.0 ± 0.0	8.8	35.0 ± 0.0	10.4	3.0	0.1	↓
Desmin	36.2 ± 0.0	10.1	36.3 ± 0.1	11.6	3.1	0.1	↓

**Table 3:** Percentage of normal karyotypes and mitotic indexes of OM-MSCs in P5, P8, and P11.

Passage	% Cells with normal karyotype (42, XY)	Mitotic Index
P5	85%	Normal
P8	62%	Normal
P11	58, 80%	Low

**Table 4:** Mean normalized concentration of each biomarker in the CM of OM-MSCs (mean ± SEM).

Biomarker	Mean normalized concentration
IL-4	0.34 ± 0.11
IL-2	0.4 ± 0.22
IL-6	0.32 ± 0.3
IFN-*γ*	0.07 ± 0.1
IL-18	0.08 ± 0.10
MCP-1	0.13 ± 0.9
TNF-*α*	0.31 ± 0.04
VEGF	0.19 ± 0.13

## Data Availability

The data that support the findings of this study are available from the corresponding author on request.

## Abbreviations

BSA: Bovine serum albumin
CFUs: Colony forming units
CM: Conditioned medium
CT: Culture time
Ct: Threshold cycle
DAB: Diaminobenzidine
FBS: Fetal bovine serum
HIER: Heat induced epitope retrieval
IFN-*γ*: Interferon-*γ*
IL: Interleukins
ISTC: International society for cellular therapy
MCP-1: Monocyte chemoattractant protein-1
MSCs: Mesenchymal stem/stromal cell
OM: Olfactory mucosa
OM-MSCs: Olfactory mucosa mesenchymal stem/stromal cell
PBS: Phosphate-buffered saline solution
PDN: Population doubling number
PDT: Population double time
PIER: Proteolytic induced epitope retrieval
RQ: Relative quantification
RT-PCR: Reverse transcriptase polymerase chain reaction
TNF-*α*: Tumor necrosis factor-*α*
VGEF: Vascular endothelial growth factor
ISCT: International society for cellular therapy.

## Supplementary Materials

Supplementary Material Table S1 represents the list of genes under study with the respective amplicon context sequences, ensemble gene ID and PCR product lengths. Table S1- List of genes under study with respective amplicon context sequence, ensemble gene ID and PCR product length.
