# Supplementary material for: Rat Olfactory Mucosa Mesenchymal Stem/Stromal Cells (OM-MSCs): A Characterization Study
Source: Int J Cell Biol. 2020 Jan 29;2020:2938258. doi: 10.1155/2020/2938258 (PMC7212324; doi:10.1155/2020/2938258)
Supplement: Supplementary Material — Table S1 represents the list of genes under study with the respective amplicon context sequences, ensemble gene ID and PCR product lengths. Table S1- List of genes under study with respective amplicon context sequence, ensemble gene ID and PCR product length. [file 2938258.f1.pdf]

Supplementary Material:

The Table S1 represents the list of genes under study with the respective amplicon context sequences, ensemble gene ID and PCR product lengths.

S1 Table - List of genes under study with respective amplicon context sequence, ensemble gene ID and PCR product length.

Marker Type		Marker	Gene Symbol	Amplicon Context Sequence	Ensembl Gene ID	PCR product
Housekeeping		GAPDH	Gapdh	TGATGGCAACAATGTCCACTTTGTCACAAGAGAAGGCAGCCCTGGTA<br>ACCAGGCGTCCGATACGGCCAAATCCGTTCACACCGACCTTCACCAT<br>CTTGTCTATGAGACGAGGCTGGCACTGCACAAGAAGATGCGGCTGTC<br>TCTA	ENSRNOG00000018630	115 bp
Multipotency		Nanog	Nanog	GAAAATTATTCTTGCTTACAAGTGTCTGCTACTGAGATGCTCTGCACA<br>GAGACTGCCTCTCCTCCGCCTTCCTCTGGGGACCTACCTCTTCAAGAT<br>AGCCCTGATTCTTCTAGCAAT	ENSRNOG00000008368	87 bp
		Oct4	Pou5f1	GCAGCTTGGCAAACTGCTCTAGCTCCTTCTGCAGGGCTTTCATATCCT<br>GGGACT CCTCGGGACTAGGTTCCACCTTCTCCAACTTCACGGC	ENSRNOG00000046487	61 bp
		Sox2	Sox2	GCAGTACAACTCCATGACCAGCTCGCAGACCTACATGAACGGCTCGC<br>CCACCTACAGCATGTCCTACTCGCAGCAGGGCACCCCCGGTATGGCG<br>CTGGGCTCCATGGGCTCTGTGGTCAAGTCCGAGGCCAGTTCC	ENSRNOG00000012199	106 bp
MSCs		CD105	Eng	AAGTCTACAAGACTGTCTCCATGCGCCTGAACATCGTCAGTCCTGACC<br>TGTCTGGCAAAGGCCTCGTCCTGCCCTCTGTACTGGGCATCACCTTTG<br>GTGCCTTCCTCATTGGGGCCCTGCTCACAGCTGCACTCTGGTACATCT<br>ATTCTCACACACGTGAGTATCCCAAGCCTCTCCACCCCCTTCCTACAG<br>TGAGTGCTCAGGGCCGGTCCACACCACCCCAGGGCA	ENSRNOG00000050190	198 bp
		CD109	Thy1	TTGGACTGTGTCTGTGCCTGGTATAGTGATACTCTGTTGAAAGTATCG<br>CCCAGTAGGCAGGGAGGGGGAGGGGCCTCAAGATAGGAGAGCGCT<br>GCTGAGCCTTTGTGGACCAAACAATAAGGATGAGAACTTAGATTGTA	ENSRNOG00000006604	110 bp
		CD73	Nt5e	ACTTCCTATGGAAATCCCATTCTTCTGAACAGCACCATTCGTGAAGAT<br>GCAGCCATCAAAGCAGACATTAACCAGTGGAGGATAAAATTAGATA<br>ATTATTCTACACAGGAACTCGGGAG	ENSRNOG00000011071	89 bp
		CD44	CD44	ATGTAGCCTTCTGGGGTGCTCTTCTCAATGGTGGATCCACTGCTGACA<br>TCCTCATCTATAATGTTTGAGGCATCGATGTCTTCTTGGTGTGTTCTAT<br>ACTCGCCCTTCTTGCTGTA	ENSRNOG00000006094	86 bp
Hematopoietic		CD45	Ptprc	CTCTCTTTGCTCATCTCTAGTTCATGCTTAAGTGGCACTCTGTTAAAGT<br>CATATGGAACAACGTTAGAACTCCTGTTTTTCTTTTTATTTTCTTCTTGA<br>TTTCCAATGTGCTGTGTCCTCCAGCTCCTGTATGAAGGAAGTCTCTGG<br>TAC	ENSRNOG00000000655	120 bp
		CD34	CD34	AACCGTCACACTGCCTACTACTTCAGACAGTACTTCTACTCCAGAAGT<br>GGAACACTCCACCTGGAACACCAGCCTGCCGTCTGTCAATGTTTCTGA<br>TTATTCACCTAATAATGTCAGCTCTGAGATGATATCAACCACTGAG	ENSRNOG00000050078	112 bp
Hematopoietic	Osteogenic	BSP	Ibsp	TCCCCGCGAGGCTCCCCGTTGTTCTCGTCATAGGTTTCATACGCAGTG<br>TTGTACTCGTTGCCTATTTGTTCGTATTCTTCCCCATACTCAACCGTGCT<br>GCTCTTTCTGGCTGG	ENSRNOG00000002158	83 bp
		Runx2	Runx2	CACACGTGTAGTAAAGGCTCAACCATGACACGTACAGCCTAGGAAC<br>ACAGATGCATTAGGAGTCTGCACCCCCCCCCCAGCCTTACCACACAG<br>CCATTCAGGGG	ENSRNOG00000020193	74 bp
	Chondrogenic	Coll II	Col2a1	CAAGGCCTTCTTGAGGTTGCCGGCTGCTTCGTCCAGGTAGGCAATGCT<br>GTTCTTACAGTGGTAGGTGATGTTCTGGGAGCCCTCAGTGGACAGTA<br>GACGGAGG	ENSRNOG00000022282	73 bp
		Aggrecan	Acan	CCCCTACCCTTGCTTCTCCAGAAGGGTCAGGAGAAACAGAGTCAACC<br>GCTGCAGACATTGATGAGTGCCTCTCAAGCCCTTGTCTGAATGGAGC<br>CACCTGCGTGGATGCCTTGGACACTTTCACATGCTTATGCCTTCC	ENSRNOG00000028992	109 bp
	Adipogenic	Ap-2	Ap2m1	AGTGCACCTGTCTCTGAGTTCTGTGGGTAGCCAAAGTCCAGAATCTCG<br>TCCAGCAGCTCGTATATGAGCACGAAATTGTTCTTGATGTTCTCCTCG<br>CTGATCTTGCCAAAGTAAGCAGCCATTACATCA	ENSRNOG00000001709	99 bp
		AdipoQ	Adipoq	TTCATGACTGGGCAGGATTAAGAGGAACAGGAGCGCTTGCAACAGT<br>AGCATCCTGAGTCCTGGAACTTTTGGTGTAATCAGATCCACTGGCACT<br>CGTACAGACAGGAATGAAG	ENSRNOG00000001821	83 bp
	Neurogenic	NGF	Ngf	TTCATGACTGGGCAGGATTAAGAGGAACAGGAGCGCTTGCAACAGT<br>AGCATCCTGAGTCCTGGAACTTTTGGTGTAATCAGATCCACTGGCACT<br>CGTACAGACAGGAATGAAG	ENSRNOG00000001821	83 bp
		GDNF	Gdnf	GGCTTTGCCAAGGACGCAGCTTTCTATCCTGGCCACTCTGAGGTGCAT<br>AGCGTAATGTCCATGTTGTTCTACACTCTGATCACAGCGTTTTTGATCG<br>GCGTACAGGCAGAACCGTACACAGATAGCAATGTCC	ENSRNOG00000016571	103 bp
	Tenogenic	Tenomodulin	Tnmd	ACTGAAGATTTGTGGACTAGTGTTTGGTATCCTGGCCTTAACTCTAAT<br>TGTCCTGTTTTGGGGGAGCAAACACTTCTGGCCCGAGGTATCCAAGA<br>AGACCTATGGCATGGAGCACACTTTCTACAGCAATGGCGAGAAGAA<br>GAAGA	ENSRNOG00000003896	116 bp
	Muscleg	Desmin	Des	ACCAGGATCTGCTCAATGTGAAGATGGCCTTGGATGTGGAGATTGCC<br>ACCTACCGGAAGCTGCTGGAGGGCGAGGAGAGCAGGATCAACCTTC<br>CGATCCAGACCTTCTCTGCTCTCAACTTCCGAGAAAC	ENSRNOG00000019810	100 bp
